# Anhedonia is associated with higher functional connectivity between the nucleus accumbens and paraventricular nucleus of thalamus

**DOI:** 10.1016/j.jad.2024.08.113

**Published:** 2024-08-26

**Authors:** Bianca T. Leonard, Sarah M. Kark, Steven J. Granger, Joren G. Adams, Liv McMillan, Michael A. Yassa

**Affiliations:** a Center for the Neurobiology of Learning and Memory, University of California, Irvine 92697, USA; b Department of Neurobiology and Behavior, University of California, Irvine 92697, USA; c Division of Depression and Anxiety Disorders, McLean Hospital, Belmont, MA 02478, USA; d Department of Psychiatry, Harvard Medical School, Boston, MA 02115, USA; e Department of Psychiatry, Oregon Health & Science University, Portland, OR 97239, USA; f VA HSR&D Center to Improve Veteran Involvement in Care, VA Portland Health Care System, Portland, OR 97239, USA; g Department of Psychiatry and Human Behavior, University of California, Irvine, CA 92697, USA

**Keywords:** Anhedonia, Paraventricular nucleus of thalamus, Nucleus accumbens, Latent factor analysis, Resting-state fMRI, Depression, Reward processing

## Abstract

**Background:**

Anhedonia stands as a life-threatening transdiagnostic feature of many mental illnesses, most notably major depression and involves neural circuits for processing reward information. The paraventricular nucleus of the thalamus (PVT) is associated with reward-seeking behavior, however, links between the PVT circuit and anhedonia have not been investigated in humans.

**Methods:**

In a sample of adults with and without psychiatric symptoms (*n* = 75, 18–41 years, 55 female), we generated an anhedonia factor score for each participant using a latent factor analysis, utilizing data from depression and anxiety assessments. Functional connectivity between the PVT and the nucleus accumbens (NAc) was calculated from high-resolution (1.5 mm) resting state fMRI.

**Results:**

Anhedonia factor scores showed a positive relationship with functional connectivity between the PVT and the NAc, principally in males and in those with psychiatric symptoms. In males, connectivity between other midline thalamic nuclei and the NAc did not show these relationships, suggesting that this link may be specific to PVT.

**Limitations:**

This cohort was originally recruited to study depression and not anhedonia per se. The distribution of male and female participants in our cohort was not equal. Partial acquisition in high-resolution fMRI scans restricted regions of interest outside of the thalamus and reward networks.

**Conclusions:**

We report evidence that anhedonia is associated with enhanced functional connectivity between the PVT and the NAc, regions that are relevant to reward processing. These results offer clues as to the potential prevention and prevention and treatment of anhedonia.

## 1. Introduction

>1 in 5 U.S. adults (22.8 %) reported experiencing a mental illness in the 2021 National Survey on Drug Use and Health conducted by the Substance Abuse and Mental Health Services Administration (Substance Abuse and Mental Health Services Administration, 2022). Current diagnostic criteria in psychiatry seem to have a limited ability to capture the reality of mental illness, including: dimensional symptom spaces, high frequency of comorbidities, and phenotypic fluctuations across time, among other features (Dalgleish et al., 2020). To address this, many researchers have begun to focus on the transdiagnostic features of mental illness, which are factors that cut across traditional diagnostic boundaries. Transdiagnostic features of mental illness may then be studied and related to the underlying neurobiology (Insel et al., 2010). One important transdiagnostic symptom is anhedonia – the diminished capacity to experience pleasure. Understanding anhedonia is critical because it is associated with increased risk of suicide, substance use disorder (specifically opioid use disorder), treatment resistance, and a reduced quality of life (Ducasse et al., 2018; Pizzagalli, 2014; Ritsner et al., 2011; Stull et al., 2022).

Anhedonia is defined as, “the inability to enjoy experiences or activities that normally would be pleasurable” according to the American Psychological Association and was first described by Théodule Ribot in 1896 (American Psychological Association, n.d.; Ribot, 1896). Anhedonia is characterized by decreased reward processing, learning, and memory. Individuals experiencing anhedonia have difficulty initiating behavior in pursuit of reward and have decreased reward responsiveness, though in-the-moment pleasure to reward is often maintained (Pizzagalli, 2010). The nucleus accumbens (NAc) is a key region in understanding anhedonia, as it is critically important to the experience of pleasure and the learning of pleasant stimuli associations(Der-Avakian and Markou, 2012). Reward prediction error via dopaminergic signaling is one well-established mechanism by which the NAc codes incentive properties of cues for learning (Schultz, 1998). Dopamine transmission is therefore important in the motivational aspects of reward that are impacted by anhedonia (Harkness et al., 2022). Meanwhile, the perception of a pleasurable stimulus is driven in part by endogenous opioids in the NAc shell (Peciña and Berridge, 2005). In humans, neuroimaging studies have characterized changes of the functional connectivity of the NAc in adults across mood disorders (Sharma et al., 2017), adolescents with anhedonia (Gabbay et al., 2013), and trauma-exposed individuals (Olson et al., 2018). While the exact cause of changes to the NAc and its circuits in anhedonia is still under investigation, the paraventricular nucleus of thalamus (PVT) is a region highly connected to the NAc that may drive the presentation of anhedonia (Moga et al., 1995; Vertes and Hoover, 2008).

The PVT is of interest to anhedonia research because across rodent studies, the PVT has been shown to play a role in processing salient experiences, broadly (Kooiker et al., 2021). More specifically, several studies in animal models examining the projections between the PVT and NAc suggest that the PVT is important for both aversive and appetitive stimulus processing (De Groote and de Kerchove d’Exaerde, 2021; Millan et al., 2017). In one study supporting the role of PVT in appetitive stimuli processing, optogenetic stimulation of oxytocin recepto-rexpressing neurons in the PVT that project to the NAc increased food-seeking behavior, providing further evidence for this projection’s role in motivation (Ye et al., 2022). Stimulation of PVT glutamatergic glucose transporter (Glut2) expressing neurons projecting to the NAc increased motivated sugar-seeking behavior (Labouèbe et al., 2016). In rats trained with an alcohol reward, lesioning of PVT➔NAcSh projections prevented context-induced reinstatement (Hamlin et al., 2009). In male rodents, the PVT stimulated dopamine release by medium spiny neurons in the NAc (Perez and Lodge, 2018).

Numerous studies have corroborated the role of PVT➔NAc projection in suppressing reward seeking behavior. The optogenetic inhibition of the PVT to NAc projection supported hedonic learning in rodents during a cocaine-conditioned place preference experiment and dopamine and methamphetamine was shown to depress excitatory postsynaptic currents from PVT inputs to the NAc (Christoffel et al., 2021). The PVT to nucleus accumbens shell (NAcSh) neurons had elevated Fos expression following opiate withdrawal in mice and their inhibition impaired the formation and expression of conditioned place aversion, providing evidence for a role in opiate and aversive memory mediation by this projection (Zhu et al., 2016). Activation of corticotropin releasing factor (CRF) producing neurons that project PVT → NAc decrease reward seeking and promote threat avoidance (Engelke et al., 2021). Inhibition of mouse PVT afferents to the NAc led to increased responses rates on an operant task regardless of reward availability, while activation of this projection reduced responses in an operant task where reward was constantly available (Lafferty et al., 2020). This suggests the PVT-NAc projection plays a key role in behavioral suppression and reward seeking suppression. Similarly, inhibition of anterior PVT → NAc projections increased cue-induced sucrose seeking in absence of a reward, only (Do-Monte et al., 2017). The discrepancy between the role of PVT-NAc projections in both appetitive and aversive behavior may be explained by the positive feedback that the PVT is providing to the NAc. Appetitive behavior may be driven by PVT projections to D1 neurons in the NAc, while aversive behavior may be influenced by feedforward inhibition via D2 projections. Indeed, this positive feedback mechanism is thought to describe how the PVT mediates motivational conflict (McNally, 2021). Biological evidence supports multiple information streams between PVT-NAc, as two projections that differ in D2 receptor expression have opposing functions (Beas et al., 2024). Sex differences have been observed in the manifestation of anhedonia induced by early life adversity (ELA) (Levis et al., 2021). ELA leads to sex-dependent activation PVT, which may result in anhedonia in males and increased reward-related behavior in females later in life (Kooiker et al., 2023). Despite the growing research on the PVT and NAc in the context of hedonic experiences, no study to date has addressed the connection between the PVT and NAc in human anhedonia.

While the PVT has been circumscribed in humans using post-mortem histological techniques, studies of the PVT in humans in-vivo have not been possible until recently. Our group has delineated the functional connectivity of the PVT in adults from the Human Connectome Project (HCP) dataset using 3D atlases that have characterized thalamic subnuclei and are employed here. The human PVT was found to be functionally connected to NAc, amygdala, and other analogous nodes to those found in the rodent literature (Kark et al., 2021). In this study, we assessed whether anhedonia is associated with PVT circuitry using functional connectivity measures derived from resting state fMRI. We hypothesized that changes in the PVT-NAc circuit, but not circuits from other midline thalamic nuclei, will be associated with anhedonia symptoms.

## 2. Methods

### 2.1. Participants

Seventy-five participants (55 female, 20 male) were recruited from the community and completed resting-state functional magnetic resonance imaging (rsfMRI). Twelve participants were excluded; ten participants had poor imaging data quality assessed in the imaging preprocessing pipeline and described more in detail below. Two participants had incomplete neuropsychiatric evaluations and were therefore excluded from the study. This yielded a total *n* = 63 (females, *n* = 48; males, *n* = 15) who were included in the study. Our previous work in this sample focused on latent anxiety in the specific diagnosis of clinical depression (Granger et al., 2022). However, because this study’s focus is anhedonia, a transdiagnostic symptom, we chose to focus on the latent anhedonia factor across groups of individuals either experiencing psychiatric symptoms (PS) or not experiencing psychiatric symptoms (NPS). While our approach was largely a dimensional approach, the breakdown of the two groups is intended to reflect severity. We hypothesized that since anhedonia symptoms are higher in the PS group than the NPS group, PVT-NAc connectivity would be more severely impacted in this group. These categories were designated following completion of the full Structured Clinical Interview for the DSM-5 Research Version (SCID-RV). The NPS group (*n* = 16) were participants who had no history of psychopathology or clinical diagnosis. The PS group (*n* = 47, females *n* = 36, males, *n* = 11) included participants with a current or past history of depression, anxiety, and obsessive-compulsive disorders. Participants with schizophrenia were excluded from the study, as schizophrenia self-reports indicate that emotional experiences are normative when exposed to positive stimuli in this condition, though individuals with schizophrenia endorse anhedonia on questionnaires. This is also known as the “anhedonia paradox, “and additional neuroimaging results suggest that the neurobiology of anhedonia in schizophrenia may differ from other psychiatric diagnoses (Dowd and Barch, 2010; Pizzagalli, 2010). Statistical analysis using two-sample *t*-tests showed no significant difference between male and female participant age (females mean age = 23 years ± 4.9, males mean age = 24 years ± 5.5, *t* = 0.−45, *p* = 0.65), Beck Depression Inventory (BDI) Total Score (females mean BDI Total Score = 26 ±15, males mean BDI Total Score = 22 ± 15, *t* = −0.83, *p* = 0.42), or BDI Anhedonia Subscores (BDI-Anh) (females mean BDI-Anh = 3.0 ± 2.3, males mean BDI-Anh = 2.7 ± 2.3, *t* = 0.45, p = 0.65).

### 2.2. MRI acquisition

MRI data were collected on a 3 Tesla Philips scanner. The imaging sequence included a high resolution structural MPRAGE (0.55 mm isotropic resolution; 273 sagittal slices, field of view = 240 × 240 mm, flip angle = 9, TR/TE = 13/5.9 ms, matrix size = 448 × 448, inversion pulse TI = 1110 ms). SENSE parallel imaging was used in two directions (2 × 1.5). The SAR (<10 %) and PNS (<75 %) were within required limits based on the scanner-calculated values. Briefly, these data were collected with a high-speed EPI single-shot pulse sequence (1.5 mm isotropic, 19 oblique axial slices parallel to the principal axis of the hippocampus, field of view = 96 × 96 mm, flip angle = 70, SENSE parallel reduction factor = 2, TR/TE = 1500/30 ms, matrix size = 64 × 64).Slices were acquired as a partial axial volume and capturing the majority of the brain with the exception of portions of the dorsolateral prefrontal cortex and superior parietal cortex. All the major networks interconnected with PVT in past work (Kark et al., 2021) was within the field of view.

### 2.3. Image preprocessing

Neuroimaging data were preprocessed and denoised by scan type using CONN toolbox version 21.a (Whitfield-Gabrieli and Nieto-Castanon, 2012) implemented in MATLAB 2019b and Statistical Parametric Mapping [SPM12, Wellcome Trust Centre for Neuroimaging, London, United Kingdom]. Structural images were centered, aligned, segmented, and normalized to the Montreal Neurological Institute (MNI) template. The functional images were realigned and unwarped (subject motion estimation and correction), centered and aligned to the AC-PC line, slice-timing corrected, segmented and MNI-normalized. Structural and functional data were resampled to 1mm^3^ and 2mm^3^ voxels, respectively. Outlier volumes were detected using Artifact Detection Tools [ART, Gabrieli Lab, Department of Brain and Cognitive Sciences, Massachusetts Institute of Technology, Cambridge, MA, USA] implemented in CONN using a threshold of motion >0.5 mm/TR and a global intensity z-score of 3. A threshold of >20 % volumes detected as outliers was used to flag subjects with high residual motion for exclusion. Ten participants were subsequently excluded for excessive motion during the resting state scan (7 females), as defined by having too few valid scans or excessive average motion for analysis (1.5 IQR [fewer than 78 valid scans and/or mean motion >0.21 mm]). As previously mentioned, two additional participants had incomplete neuropsychiatric evaluations and were excluded.

Denoising consisted of the six realignment parameters and their first-order derivatives, spike regressors generated from outlier detection, and anatomical CompCorr (first five components from white matter and CSF). Preprocessed resting state scans for each participant were linearly detrended and a commonly used bandpass filter (0.008–0.09 Hz) was applied after nuisance regression (RegBP).

### 2.4. ROI-to-ROI semipartial correlation analysis

**Fig. 1.**
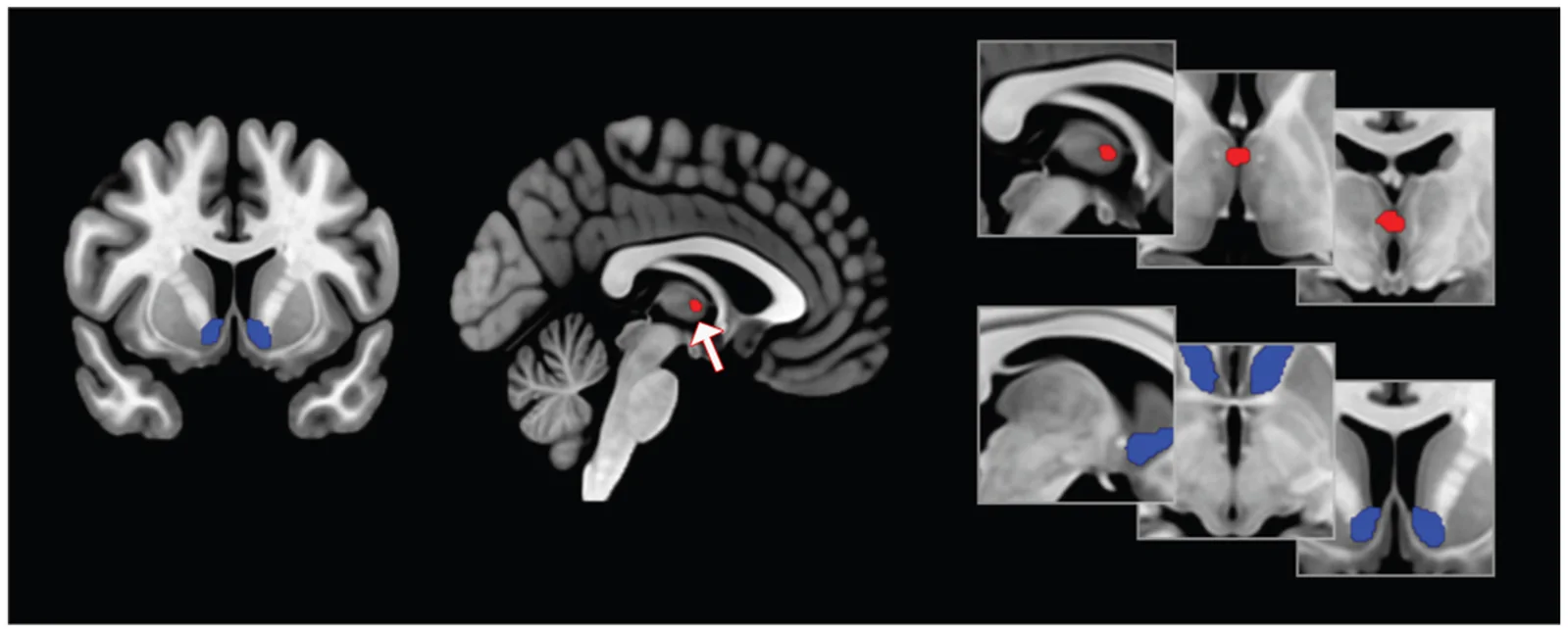
The bilateral PVT ROI (176 mm^3^) is depicted in red and the bilateral NAc ROI (1528 mm^3^) is depicted in blue. (For interpretation of the references to color in this figure legend, the reader is referred to the web version of this article.)

Following preprocessing and denoising, mean BOLD activity for each ROI was extracted using REX (http://web.mit.edu/swg/software.htm) implemented in CONN. Given the smaller size of our ROIs and proximity to the ventricles, unsmoothed BOLD time series data was used. We utilized 3D digital seed masks from the “Thalamus Atlas” of the Swiss Federal Institute of Technology (ETH) in Zurich and University of Zurich, Switzerland (Jakab et al., 2012; Krauth et al., 2010). The digital model was utilized with written consent from the Computer Vision Laboratory of the ETH Zurich. A bilateral PVT ROI was generated (176 mm^3^), as well as a bilateral midline thalamic nuclei control group, including central lateral, central medial, and parafascicular subnuclei (4369 mm^3^), which can also be found in our previous work (Kark et al., 2021). The bilateral NAc ROI (1528 mm^3^) was generated using the Brainnetomme atlas (Fan et al., 2016); https://atlas.brainnetome.org/index.html), see Fig. 1.

During the first-level analysis, the average time series of each region was extracted and the semipartial correlation was calculated as a measure of functional connectivity between pairs of regions. These values were then transformed to Fisher’s coefficients and exported. In R Studio, Spearman’s correlations between participant anhedonia factor scores and functional connectivity z-scores were calculated.

### 2.5. Anhedonia factor analysis

Five latent factors were derived from Beck Depression (BDI) and Anxiety (BAI) Inventories, as described in a previous study (Granger et al., 2022), using the “Psych” toolbox in R (https://rstudio.com). This study used the “anhedonia” factor which included questionnaire items describing, “loss of pleasure, loss of interest, loss of energy, punishment, crying, lack of concentration, and tiredness.” The summation of these items resulted in an anhedonia factor score for each participant (range = 0–24, mean = 9.9 ± 6.5). Factor loadings for the anhedonia factor (Factor 2) in this study are reported in Table 2 of Granger et al. (2022). This method was validated by calculating the normalized mutual information score, which reported a moderate degree of consistency between this study and previously reported results with a larger sample (Lee et al., 2018).

### 2.6. Statistical analyses

A z-score for each participan’s anhedonia factor score was calculated in R (https://rstudio.com) and then correlated with the PVT-NAc and Midline Thalamus-NAc functional connectivity z-scores described above in the ROI-ROI Semipartial Correlation Analysis section.

## 3. Results

PVT-NAc functional connectivity is positively associated with anhedonia in males with psychiatric symptoms.

**Fig. 2.**
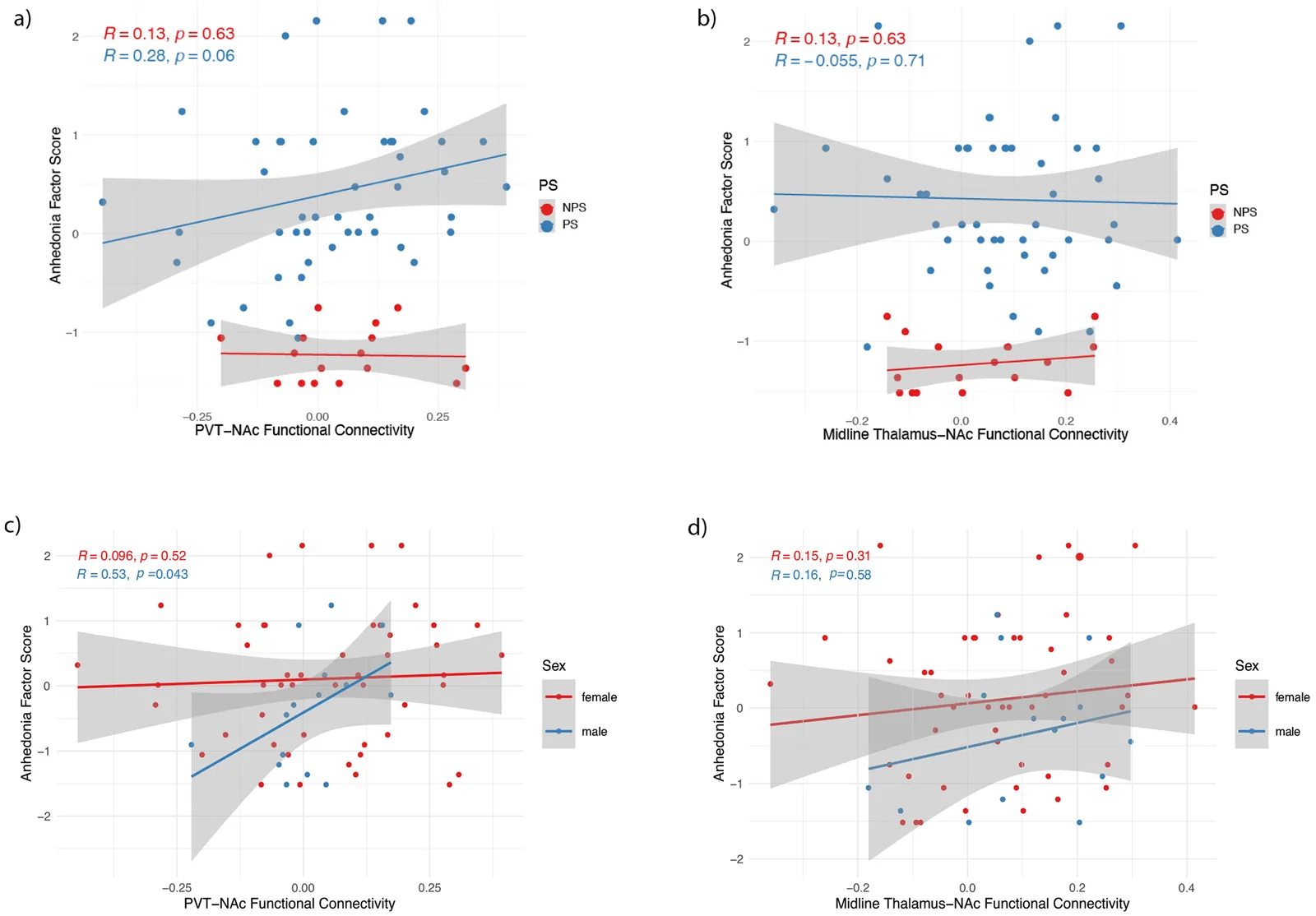
a) Association between PVT-NAc FC and b) Midline Thalamus-NAc and anhedonia stratified by participants experiencing psychiatric symptoms (PS) and those without (NPS). c) Association between PVT-NAc FC and d) Midline Thalamus-NAc and anhedonia in all participants, stratified by sex.

We found no significant correlation between anhedonia factor scores and PVT-NAc FC across the entire sample (Supplemental Fig. S1a, *R* = 0.15, *p* = 0.25, *n* = 63), however, when the sample was divided into PS and NPS groups, we found that PS participants had a positive trending association between PVT-NAc FC and anhedonia (Fig. 2a, R = 0.28, *p* = 0.06, *n* = 47). We did not find a similar relationship in the NPS group (Fig. 2a, R = 0.13, *p* = 0.63, *n* = 16). In a control comparison, we investigated whether midline thalamus-NAc FC was associated with anhedonia factor scores and found no significant correlations for either NPS (Fig. 2b, R = 0.13, p = 0.63, n = 16) or PS groups (Fig. 2b, R = −0.055, *p* = 0.71, n = 47).

Sex-stratified analysis of PVT-NAc FC and Anhedonia factor scores revealed a positive relationship in males (Fig. 2c, R = 0.53, *p* = 0.043, *n* = 15), but not in females (Fig. 2c, *R* = 0.096, *p* = 0.52, *n* = 48). The difference in the two correlations did not reach statistical significance. Midline thalamus-NAc FC was not associated with anhedonia factors scores in either sex (Fig. 2d, females: R = 0.15, *p* = 0.31, n = 48; males: *R* = 0.16, *p* = 0.58, n = 15).

**Fig. 3.**
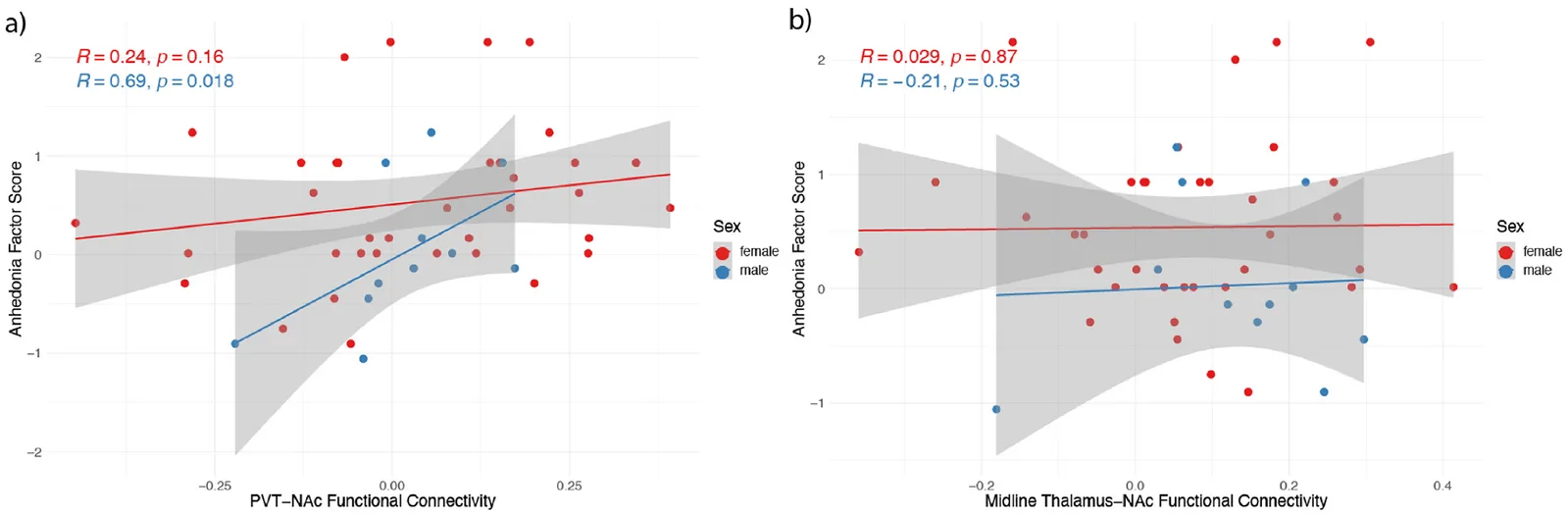
a) Association between PVT-NAc and anhedonia and b) association between Midline Thalamus - NAc in participants with psychiatric symptoms and stratified by sex.

Restricting our sample to the PS participants increased the strength of the male PVT-NAc and anhedonia relationship (Fig. 3a, R = 0.69, *p* = 0.018, *n* = 11), while the association in females did not reach significance (Fig. 3a, R = 0.24, *p* = 0.16, *n* = 36). Midline thalamus-NAc FC did not correlate with anhedonia scores in either sex in this subgroup (Fig. 3b, females: *R* = 0.029, *p* = 0.87, n = 36, males: *R* = −0.21, *p* = 0.53, n = 11).

## 4. Discussion

In this study, we tested the hypothesis that anhedonia symptoms in adults are associated with functional connectivity between the PVT and the NAc. Consistent with our prediction, we found that greater anhedonia scores were associated with elevated functional connectivity between the PVT and the NAc in male participants who reported active psychiatric symptoms. To our knowledge, this is the first paper to examine rsFC between the PVT and NAc in adults with anhedonia.

Several past studies have reported changes of NAc rsFC in anhedonia. In an adult female cohort with PTSD, anhedonia scores were associated with decreased functional connectivity in the left NAc seed to a cluster that corresponded to left caudate and part of the thalamus (Pessin et al., 2021). Another study demonstrated that anhedonia is positively associated with NAc to medial prefrontal cortex (mPFC) rsFC in trauma-exposed participants (Olson et al., 2018). In major depressive disorder (MDD), which often requires anhedonia for clinical diagnosis (Tolentino and Schmidt, 2018), NAc rsFC to ventromedial prefrontal cortex and hippocampus was negatively associated with appetite, though functional connectivity measures did not successfully distinguish between MDD and control participants (Kroemer et al., 2022). However, another study showed that patients with MDD had decreased rsFC between the NAc and reward, executive, salience and default mode networks compared to controls (Zhou et al., 2022). While our study and past work demonstrate that functional connectivity changes of reward circuits are implicated in anhedonia, the factors shaping the development of these networks are unknown.

Early life adversity (ELA) is one form of chronic stress understood to shape neural circuit development across the lifespan, and it may give rise to anhedonia. ELA seems to have a sex-specific effect on the development of anhedonia (Birnie et al., 2022; Kangas et al., 2021; Levis et al., 2022). In males, the limited bedding-and-nesting (LBN) model of ELA produced anhedonia towards sucrose and social play, as well as lowered the hedonic setpoint of cocaine administration (Bolton et al., 2018). In females, the LBN model resulted in elevated consumption of highly palatable foods and opiates (Levis et al., 2021). The LBN model of ELA has been shown to strongly activate neurons of the PVT, confirming that this region is tied to the early life environment, changes in salient information processing, and neuropsychiatric disorder risk (Kooiker et al., 2023, 2021). The PVT may therefore influence motivated behaviors that manifests as anhedonia through its connections to the NAc and other reward processing regions including the ventral tegmental area (VTA) and mPFC.

Our study’s positive association between anhedonia and NAc-PVT rsFC seems to be driven by male participants with psychiatric symptoms. However, the difference between the sex stratified correlations fell short of significance and thus we cannot attribute this finding to a sex difference in anhedonia. This is consistent with literature that has shown that while anhedonia contributed to lower NAc volume and responsiveness to reward in a monetary incentive delay task, this effect was not moderated by sex (Wacker et al., 2009). Sex differences in the epidemiology of depression and anxiety have been observed, but neurobiological differences in the etiology of anxiety, depression, and anhedonia have not been established in humans, though preclinical, animal models suggest molecular mechanisms may underlie sex differences in stress and anhedonia (Bangasser and Cuarenta, 2021).

It is important to note that in humans, gender and sex exist on a spectrum and are related to numerous variables. The expression of an individual’s gender and sex depends on multiple factors, including: early life socialization, cultural expectations, identity, hormones, and genetics (McCarthy, 2016; Montañez, 2017). Sex differences cannot be established without careful consideration of these factors as they relate to brain, behavior, and experimental design. Previous research on fMRI sexual dimorphism has largely omitted hormonal nor chromosomal contributions to functional connectivity, though one study evaluated hormonal levels and resting-state functional connectivity in menstruating women across their cycles. The authors found that menstrual changes in hormones had no effect on resting state, although this study’s sample size may have been too low to examine brain-wide associations on rsfMRI (Hjelmervik et al., 2014). Anhedonia research could be improved by testing hormone levels of all participants, regardless of gender identity, and recruiting a more diverse cohort in terms of gender identity. Before we can draw conclusions about the effect of sex on brain development and activity, we will need to design studies that include more accurate representations of participants’ lived experiences and the complex interactions of variables that give rise to sex and gender identity.

The results of this study suggest that there is a pattern of functional connectivity between the PVT and reward processing regions that is shared among males experiencing anhedonia. This pattern may reflect an important role that the PVT to NAc communication is playing in decision-making and behavioral changes in anhedonia.

Limitations of this study are as follows: the sample size of the cohort is relatively small and resulted in an imbalance in the NPS/PS group distributions. This cohort was recruited to study depression and not anhedonia per se, so the representation of anhedonia in our participants was limited, though it is worth noting that anhedonia is considered a core feature of depression. Additionally, specific tools designed to address anhedonia such as the Snaith-Hamilton Pleasure Scale (Snaith et al., 1995) or the Dimensional Anhedonia Rating Scale (Rizvi et al., 2015), were not used here, which necessitated the use of the latent factor approach.

The distribution of male and female participants in our cohort was not equal, and this made sex differences more difficult to study. Additionally, some participants categorized as either reporting psychiatric symptoms (PS) or not (NPS) had similar anhedonia factor scores, which points to the need for better diagnostic criteria in psychiatric research and practice. Interestingly, there has been movement in the field towards more dimensional assessments of psychiatric symptoms e.g. The Hierarchical Taxonomy of Psychopathology (HiTOP) (Kotov et al., 2017), which are more consistent with the National Institute of Mental Health’s Research Domain Criteria (RDoC) (Woody and Gibb, 2015), of which anhedonia is a core element (Domain: Negative Valence Systems; Construct: Loss).

Other limitations relate to difficulties with neuroimaging research, including participants who moved too much during image acquisition, limiting the number of usable scans. While the use of high resolution in this investigation is a distinct advantage, especially for such a small region like the PVT, the partial acquisition limited our ability to examine regions outside of the regions of interest. Future work should evaluate PVT functional connectivity to other regions that are considered important in anhedonia, such as the anterior cingulate cortex and other frontal regions. PVT functional connectivity might also be evaluated in comparison to behavioral outcomes in tasks established in anhedonia literature, such as the probabilistic reward task (PRT) (Pizzagalli et al., 2008). It would also be informative to evaluate participants’ exposure to early life adversity to understand its relationship to PVT functional connectivity and anhedonia. One such tool to evaluate this would be the Questionnaire of Unpredictability in Childhood (QUIC; (Glynn et al., 2019). Understanding neurocircuitry altered in anhedonia and the risk factors for its development will allow for the innovation of prevention protocols and clinical interventions.

### 4.1. Citation diversity statement

Recent work in several fields of science has identified a bias in citation practices such that papers from women and other minority scholars are under-cited relative to the number of such papers in the field (1–9). Here we sought to proactively consider choosing references that reflect the diversity of the field in thought, form of contribution, gender, race, ethnicity, and other factors. First, we obtained the predicted gender of the first and last author of each reference by using databases that store the probability of a first name being carried by a woman (5, 10). By this measure and excluding self-citations to the first and last authors of our current paper), our references contain 12.28 % woman(first)/woman(last), 8.77 % man/woman, 29.87 % woman/man, and 49.08 % man/man. This method is limited in that a) names, pronouns, and social media profiles used to construct the databases may not, in every case, be indicative of gender identity and b) it cannot account for intersex, non-binary, or transgender people. Second, we obtained predicted racial/ethnic category of the first and last author of each reference by databases that store the probability of a first and last name being carried by an author of color (11,12). By this measure (and excluding self-citations), our references contain 14.4 % author of color (first)/author of color(last), 18.81 % white author/author of color, 18.48 % author of color/white author, and 48.30 % white author/white author. This method is limited in that a) names and Florida Voter Data to make the predictions may not be indicative of racial/ethnic identity, and b) it cannot account for Indigenous and mixed-race authors, or those who may face differential biases due to the ambiguous racialization or ethnicization of their names. We look forward to future work that could help us to better understand how to support equitable practices in science.

## Supplementary Material

supplement

Supplementary data to this article can be found online at https://doi.org/10.1016/j.jad.2024.08.113.
