# Supplementary material for: Anhedonia is associated with higher functional connectivity between the nucleus accumbens and paraventricular nucleus of thalamus
Source: J Affect Disord. Author manuscript; Available in PMC 2026 Sep 21. (PMC13593191; doi:10.1016/j.jad.2024.08.113)
Supplement: Supplementary file 1 [file NIHMS2157141-supplement-supplement.docx]

# Supplemental Figures

**Supplement Figure S1:** a) Association between PVT-NAc and anhedonia and b) association between Midline Thalamus - NAc in all participants.
